# Antimicrobial Resistance, One Health Interventions and the Least Restrictive Alternative Principle

**DOI:** 10.1093/phe/phae004

**Published:** 2024-04-23

**Authors:** Davide Fumagalli

**Affiliations:** Department of Philosophy, Linguistics and Theory of Science, University of Gothenburg, 412 55 Göteborg, Sweden

## Abstract

Antimicrobial resistance (AMR) is increasingly recognised as a threat to human, animal and environmental health. In an effort to counter this threat, several intervention plans have been proposed and implemented by states and organisations such as the WHO. A One Health policy approach, which targets multiple domains (healthcare, animal husbandry and the environment), has been identified as useful for curbing AMR. Johnson and Matlock have recently argued that One Health policies in the AMR context require special ethical justification because of the so-called *least restrictive alternative principle*. This article analyses and rejects two assumptions that this argument relies on. The first assumption is that One Health policies are generally more restrictive than their alternatives because they target more domains and impact more people. The second assumption is that the least restrictive alternative principle has a special normative importance in that it establishes a systematic presumption in favour of the least restrictive policy options. Once these assumptions are rejected, the use of One Health policies on AMR can be justified more easily than Johnson and Matlock argue.

## Introduction

Antimicrobial resistance (AMR) is a phenomenon consisting of the emergence and spread of microorganisms capable of resisting the effects of antimicrobial substances, such as antibiotics. This phenomenon causes the currently available antibiotics to be increasingly ineffective, eventually making bacterial infections more dangerous and many medical treatments relying on antibiotics (including most surgeries) riskier. AMR poses a very urgent challenge to human health and society and can endanger wildlife and the environment (Nuffield Council, 2007; IACG, 2019; WHO, 2021). The WHO and others have called for coordinated actions regarding the development of new classes of antibiotics as well as several preventive, stewardship and surveillance measures (Hoffman and Outterson, 2015; WHO, 2015; Van Katwyk et al., 2019; Jamrozik and Selgelid, 2020).

Recent research highlights the important role of the environment in driving AMR (Larsson and Flach, 2021). In certain environments such as pharmaceutical manufacturing plants, healthcare facilities and industrial farms, and in wastewater from these sources, there is a strong selective pressure that makes it more likely for bacterial populations to develop or acquire antibiotic-resistant genes (Forsberg et al., 2012). Prevention measures against the spread of antibiotic-resistant bacteria, resistance genes and antibiotic residues in the environment must therefore involve environmental considerations (Finley et al., 2013; Bengtsson-Palme et al., 2018).

It is widely recognised that battling AMR effectively requires policymakers to consider and address the environmental dimensions of this problem (Pruden et al., 2013; WHO, 2015, 2021). One salient approach is the One Health policy framework, which seeks to target not only human, animal or environmental sources of AMR in a piecemeal fashion, but also several of these domains at once. In the context of farming, for example, a One Health policy could aim at reducing not only antibiotic use in animals but also environmental pollution resulting from that use.

Any type of public health intervention against AMR, including One Health interventions, is going to need some ethical justification (Nijsigh et al., 2020). A traditional principle used in the justification of public health interventions is the *least restrictive alternative principle*, which mandates that ‘public health agents should seek to minimize the infringement of general moral considerations’ (Childress et al., 2002).^1^ This implies that if a decision maker is to decide between two public health policies, they should prioritise the alternative that would pose fewer restrictions to other relevant moral considerations, such as autonomy and privacy, other things being equal.

In a recent article, Johnson and Matlock (2022) claim that One Health AMR policies would typically be more restrictive than their alternative since they target more domains. Therefore, they argue, One Health AMR policies need special justification because of the least restrictive alternative principle. Their article then provides two arguments in favour of using One Health interventions to combat AMR, despite their being more restrictive, one appealing to the potentially greater effectiveness of such interventions, the other appealing to their capacity to accurately track moral responsibility. In this article, I will provide a critique of two assumptions underlying these arguments. Firstly, I criticise the additive notion of restrictiveness that Johnson and Matlock must assume to ground the idea that a One Health policy option would often be more restrictive than its alternatives. Secondly, I reject the idea that the least restrictive alternative principle plays a special normative role by establishing a systematic presumption in favour of the least restrictive policy option. I conclude that One Health policies addressing AMR, do not bear a special justificatory burden against the *least restrictive alternative principle.* Therefore, such policies can be more easily justified than Johnson and Matlock assume.

## Justifying the More Restrictive Alternative

In motivating their discussion of One Health AMR policies, Johnson and Matlock make two basic claims. Firstly, they argue that One Health AMR policies are often more restrictive because by targeting several domains at once, they potentially impact a wider selection of stakeholders and levels of society.^2^ Secondly, they argue that in virtue of being typically more restrictive, One Health AMR policies are in special need of ethical justification. They write:

Policies that take a ‘One Health’ approach deal with this cross-reservoir spread, but are often more restrictive concerning human actions than policies that focus on a single reservoir. As such, the burden of justification lies with these more restrictive policies. (Johnson and Matlock, 2022: 1)

Based on these assumptions, Johnson and Matlock provide one justification based on *effectiveness* and another one based on *moral responsibility tracking*. Firstly, they claim that policymakers have reason to choose the policy that will more effectively prevent the most harm and suffering to humans, animals and the environment. Therefore, they argue that a policy could be justified despite being more restrictive if it is more effective than its alternatives. Secondly, they argue that One Health policies can hold actors *accountable* for past AMR-related harms and identify those who are capable of *preventing* them more accurately than alternative policies (Johnson and Matlock, 2022).

In short, Johnson and Matlock conclude that One Health policies in the field of AMR may be justified despite being more restrictive if one or several of the following conditions are met: (i) they are proven to be more effective than single-domain policies, (ii) they can be more effective at assigning accountability for past actions, or (iii) they would be more effective at reducing future risk of harmful behaviour. In order to establish if these conditions hold, they argue, it is crucial to rely on empirical evidence.

Johnson and Matlock successfully identify AMR as a pressing issue and highlight the role of the environment as a driver of resistance. Moreover, their article contributes to the as yet very limited public health ethics literature highlighting ethical aspects of AMR in the environment and related policies (Nijsingh et al., 2019, 2020; Malmqvist and Munthe, 2020; Munthe et al., 2021; Malmqvist et al., 2023). Importantly, the authors make the case for the inclusion of more multidisciplinary considerations in normative policymaking and the need for further empirical research on which specific interventions could be the most effective. Despite these advantages, Johnson and Matlock’s argument rests on two basic assumptions that I will argue are implausible.

## The Restrictiveness of One Health Policies

This section will challenge the assumption that One Health policies, by targeting more domains, are typically more restrictive than alternative policies targeting single domains—the assumption that motivates the need for special justification in light of the *least restrictive alternative principle*. Two examples in public health policy will support my claim that One Health policies can only really be considered generally more restrictive if we are relying on an implausible interpretation of restrictiveness.

As already mentioned, Johnson and Matlock argue that One Health policies are typically more restrictive because of their expansiveness, because they typically target more societal levels and stakeholders than single-domain policies. Considering One Health policies more restrictive simply in virtue of this expansiveness, however, relies on an implausible interpretation of the notion of restrictiveness, which I will call *additive restrictiveness*. On this interpretation, the restrictiveness of a policy is determined by adding together all the individual instances of restriction that affect a population. Restrictiveness is considered as a function of the number of individuals hit by the intervention and the impact those interventions have on them. When operating with this interpretation of restrictiveness, the *least restrictive alternative principle* is likely to call for special justification of policies that affect a larger amount of people or a wider set of domains.

In contrast, *individual restrictiveness* evaluates the restrictiveness of a policy in virtue of how much that policy affects the individuals that are impacted the most. This means that restrictiveness is a function of the restrictions a policy imposes on single individuals, irrespectively of how many are targeted by the intervention. When operating with this interpretation of restrictiveness, the *least restrictive alternative principle* will mandate the policy that is least restrictive for the individual who is restricted the most by that policy.

Operating with the notion of *additive restrictiveness* in the context of public health leads to counterintuitive results. Consider the following two examples. The first is the case of an intervention that aims at reducing the spread of a dangerous infectious disease. Suppose that one could intervene on this issue by making it compulsory to wear a mask on public transport. An equally effective alternative policy would instead mandate coercive isolation of a few infected individuals. These interventions would definitely restrict people’s lives differently. The first policy would impact a very large number of people to a small degree, while leaving many aspects of their lives unchanged. The second policy would have a much more severe impact on each isolated individual but affect very few people. Comparing these two options, the first one would be the most restrictive if we applied the notion of additive restrictiveness, while the second would be the most restrictive if we were operating with the notion of individual restrictiveness. Clearly, in this case, it makes more sense to understand restrictiveness in an *individual* not *additive* sense, such that the coercive isolation policy is the most restrictive one.

The second example is the case of AMR arising in wastewater as a result of pharmaceutical use by human patients. Suppose one could intervene with a preventive non-One Health approach which significantly reduces medically indicated antibiotic prescriptions for a limited patient population. This solution would likely limit patients’ access to medicines, potentially lowering the quality of treatment they receive. Patients would struggle with infection longer and face an increased risk of related complications. Alternatively, one could intervene with a multiple-domain approach, requiring water service providers to upgrade wastewater treatment plants to better remove pharmaceutical residues, incentivizing the pharmaceutical industry to invest in long-term research and development on less polluting antibiotics and making some changes in prescription practices but more modest ones than on the first alternative. The first approach would be the more restrictive one from an individual point of view, whereas the second one would be more restrictive in an additive sense because it would be financially costly necessitating increased water fees or taxes imposing a small but notable economic burden on very many people. Intuitively, the first option clearly seems more restrictive in the sense relevant to the *least restrictive alternative principle*.

These two examples suggest that the restrictiveness of a policy cannot be simply estimated by adding together the individual instances of restriction that the policy imposes across domains. The assumption of *additive restrictiveness* is too insensitive to how each specific individual is affected. Hence, I reject Johnson and Matlock’s assumption that the expansiveness of One Health policies is likely to make them more restrictive in a sense relevant to a plausible interpretation of the *least restrictive alternative principle*. In fact, considering the specific case of AMR, the second example suggests that One Health policies may typically be *less* restrictive than single-domain policies because they distribute the burden of policies across more people.

## The Role of the Least Restrictive Alternative Principle

This section challenges the normative role that Johnson and Matlock assign to the *least restrictive alternative principle*. They write:

We accept an initial presumption in favor of the least restrictive alternative among the policies available, and as such, that the burden of justification for ethically preferring a more restrictive policy lies squarely with that more restrictive policy and the other moral concepts that might, together, justify preferring it instead. (Johnson and Matlock, 2022: 3)

Johnson and Matlock here appear to assume that when evaluating policy alternatives, the least restrictive alternative principle has a special role compared to other moral principles. More precisely, it establishes, at the outset of the analysis, a presumption against whatever policy alternative is more restrictive than the least restrictive one. This places the burden of proof (or burden of justification) on proponents of the more restrictive alternative to point to other moral considerations (e.g. effectiveness) that must be weighty enough to justify overturning the starting presumption. However, if these considerations are not sufficiently compelling, the presumption stands and policymakers must choose the least restrictive option.

This interpretation of the *least restrictive alternative principle* contrasts with how the principle is commonly understood in authoritative sources. For instance, Childress and colleagues write:

Even when a proposed policy satisfies the first three justificatory conditions—that is, it is effective, proportionate, and essential in realizing the goal of public health—public health agents should seek to minimize the infringement of general moral considerations. (Childress et al., 2002: 173)

In this interpretation, the *least restrictive alternative principle* is used to choose between similarly effective, proportionate and essential alternatives, which carry different risks of infringing on personal liberties to promote public health. Given two alternative policies that are similarly effective in achieving their goal, proportionate regarding their imposed burdens and benefits, and where either one or the other must be applied to reach the goal, the preferable option is the one that is less intrusive and infringes less on individual autonomy. As a second example consider Kass:

If 2 options exist to address a public health problem, we are required, ethically, to choose the approach that poses fewer risks to other moral claims, such as liberty, privacy, opportunity, and justice, assuming benefits are not significantly reduced. (Kass, 2001: 1780)

Finally, consider a similar formulation of the principle, provided by Persad:

A state or local public health agency shall employ the least restrictive alternative in the exercise of its authorities or powers, especially compulsory powers. This means that where the agency may exercise one or more of its authorities or powers to accomplish essential public health services and functions, it shall, to the extent possible, employ the policy or practice that least infringes on the rights or interests of individuals. Employing the least restrictive alternative does not require the agency to adopt policies or programs that are less effective in protecting the public’s health or safety. (Persad, 2021: 182)

In these sources, the function of the *least restrictive alternative principle* is not to establish an initial presumption. Rather, it plays a more modest checks-and-balances role, guiding choices between options that are comparable or similar with respect to other moral considerations (e.g. effectiveness). By contrast, Johnson and Matlock’s talk of a presumption suggests that they assign greater normative importance to the principle.

However, the idea of a presumption can be interpreted in different ways. In one interpretation, which is common in legal contexts, the standards of evidence that must be satisfied to establish a presumption are lower than the standards of evidence that must be satisfied to overturn it (Koplin and Selgelid, 2015). So, in the case under discussion, proponents of the least restrictive policy alternative would not need as compelling evidence to support their case as proponents of a more restrictive but more effective policy. The more restrictive alternative would systematically be harder to justify (in terms of producing evidence), despite it being more effective. Such asymmetrical standards of evidence make sense in criminal law because they track an underlying normative asymmetry. The notion ‘innocent until proven guilty’ expresses an asymmetry in standards of evidence that reflects the recognition that punishing the innocent is normally morally worse than failing to punish culpable offenders. However, it is unclear whether similar underlying normative asymmetries are present in the context of public health, including AMR policy (cf. Koplin and Selgelid, 2015). It is unclear that protecting personal freedoms is systematically more important than protecting people from drug-resistant infections in a way that justifies holding those who defend any AMR policy that is more restrictive than the least restrictive one to higher standards of evidence than those who oppose it. At the very least, establishing such an underlying normative asymmetry would require some principled argument that Johnson and Matlock do not provide.

But perhaps this is not what Johnson and Matlock have in mind when they talk of a presumption. In an earlier section of the article, they write:

We hold to a pluralist approach (Dawson, 2016), claiming that which policy is ethically preferable will depend on a number of different factors, such as how restrictive it is, how effective it is, whether it is proportionate, whether it distributes burdens in an acceptable way etc. What’s more, these values may trade off against each other[…]. (Johnson and Matlock, 2022: 1)

This appears to suggest that public health policies should be evaluated based on several different ethical considerations, where each has *pro tanto* weight but none plays a special role in decision-making. This view is consistent with only an implausibly weak interpretation of the idea of a presumption in favour of the least restrictive alternative. It does imply that other things being equal, one should choose the least restrictive policy available. But it also implies that other things being equal, one should choose the most effective policy, or the one that distributes burdens most equitably. Since all plausible moral principles play the same role on this approach, it is potentially misleading to single out any particular principle (e.g. the least restrictive alternative principle) as establishing a presumption.

So, depending on how we understand it, the notion of a presumption in favour of the least restrictive alternative seems either inappropriate in the context of AMR policy or potentially misleading.

## Conclusion

I have challenged Johnson and Matlock’s claim that One Health policies on AMR need special justification against the *least restrictive alternative principle* in two ways. Firstly, I have questioned the implicit assumption of additive restrictiveness, which is assumed by their claim that the expansive nature of One Health policies typically makes them more restrictive. I have argued that an individual notion of restrictiveness is more suitable for evaluating the degree of restrictiveness of public health policies and that One Health policies are not typically more restrictive in this sense.

Secondly, I have questioned Johnson and Matlock’s suggestion that the *least restrictive alternative principle* has special normative importance in a way that justifies a systematic presumption in favour of the least restrictive policy option. It is more plausible to think of the principle as one among several prima facie principles that policymakers must balance against each other case by case when determining what to do.

In conclusion, justifying the use of One Health policies in the AMR context is considerably easier than Johnson and Matlock argue.

## Footnotes

1 This principle has also been referred to as the *least infringement principle* (Childress et al., 2002), *minimisation of burden principle* (Kass, 2001) and *least restrictive alternative principle* (Persad, 2021).

2 It should be noted here that the authors are aware that in some cases One Health policies are less restrictive than their alternatives and, in such cases, ‘the ethical justification may be less necessary, as there is less of a presumption against One Health policies to begin with’ (Johnson and Matlock, 2022: 4). Even with this caveat the principal assumption they rely on is that, typically, a One Health-type policy is going to be more restrictive than its alternatives.
